# Supplementary material for: Rapid and continuous regulating adhesion strength by mechanical micro-vibration
Source: Nat Commun. 2020 Mar 27;11:1583. doi: 10.1038/s41467-020-15447-x (PMC7101336; doi:10.1038/s41467-020-15447-x)
Supplement: Supplementary file 1 — Supplementary Information [file 41467_2020_15447_MOESM1_ESM.pdf]

Rapid and continuous regulating adhesion strength by mechanical vibration

Shui et al.

Supplementary Figures

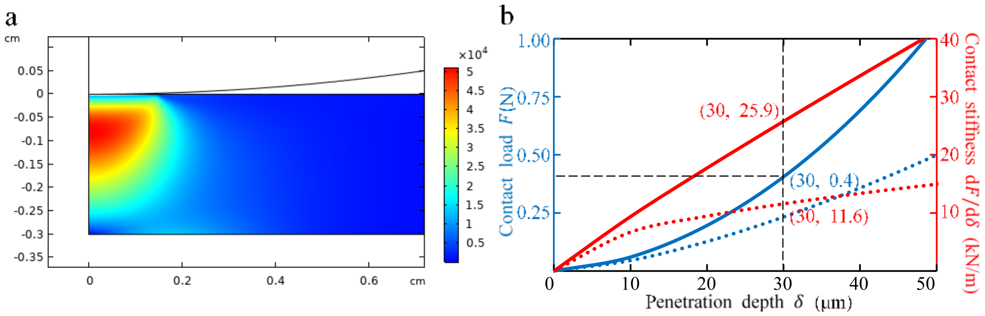

Supplementary Figure 1. The effect of platform thickness on PDMS-glass contact behaviour. (a) Distribution of the contact von Mises stress (Pa), $\delta = 30$ μm. (b) The contact load (blue curves) and stiffness (red curves) versus δ (the solid lines correspond to a base with a thickness of 3 mm, and the dotted lines correspond to a base with an infinite thickness).

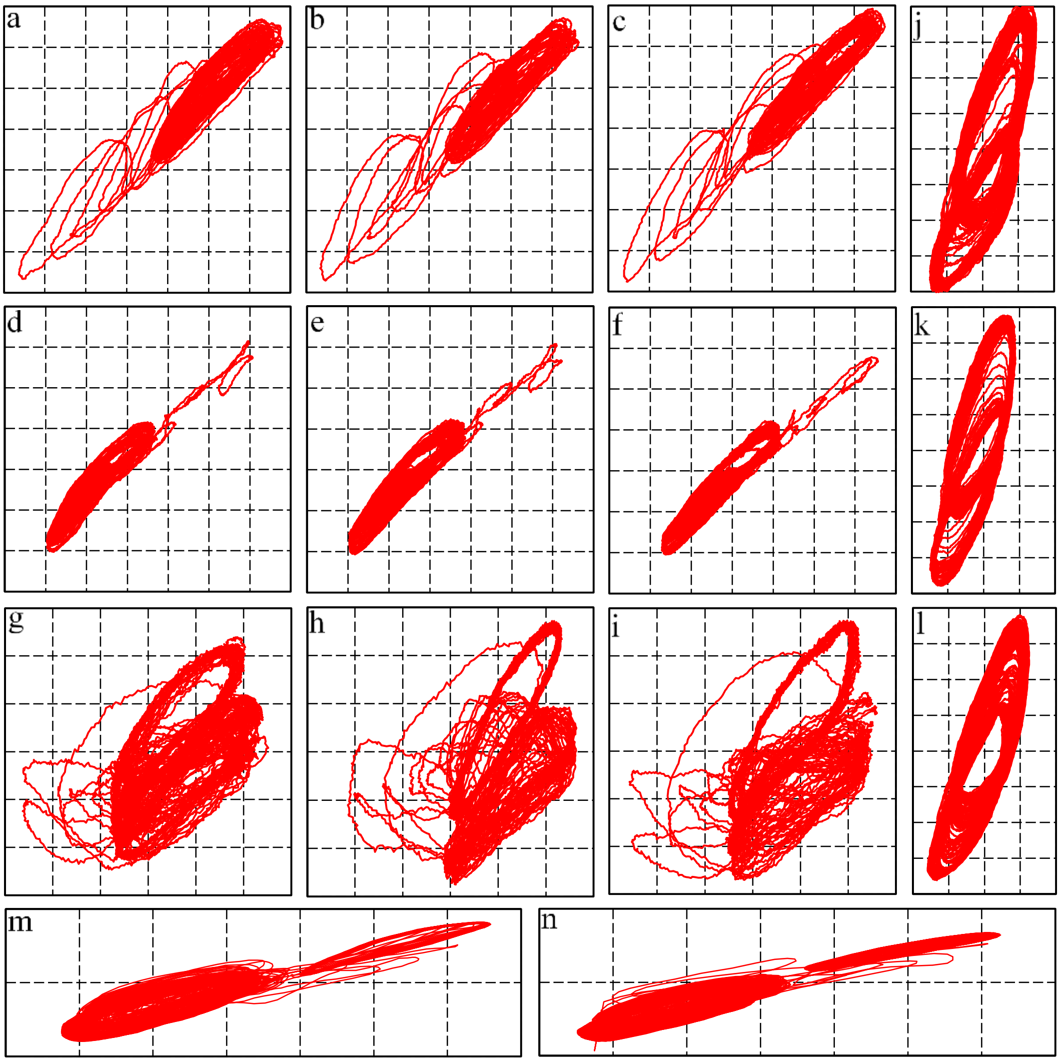

Supplementary Figure 2. Displacement of the contactor versus displacement of the platform under different conditions. (a)-(c) the abrupt increase in actuation amplitude. (d)-(f) The abrupt decrease in actuation amplitude. (g)-(i) The abrupt increase in actuation frequency. (j)-(l) The abrupt increase in apparent contact load. The corresponding results are shown in **Supplementary Table 1**. The vertical axis are δ, and the horizontal axis are $A_b \cos \omega t$. The displayed dimensions of the coordinate axes are 0.28×0.28 mm^2 in (a)-(f), 0.12×0.12 mm^2 in (g)-(i), 0.16×0.32 mm^2 in (j)-(l), and 0.28×0.08 mm^2 in (m)-(n).

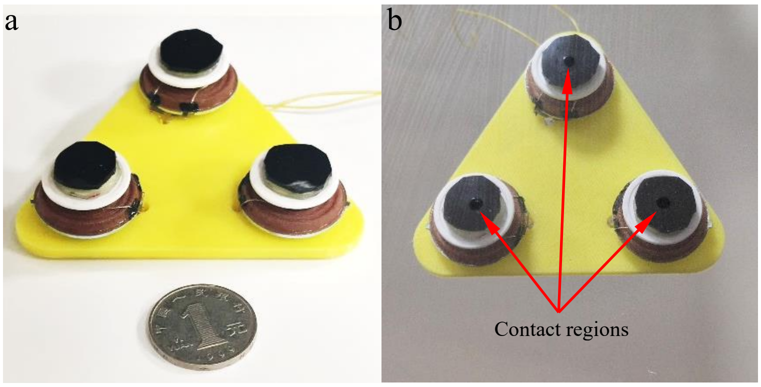

Supplementary Figure 3. The demo for regulating the adhesion between a flat glass wall and a designed contactor. (a) View of the designed contactor. (b) Its contact state with a flat glass in the demo. The designed contactor (with a total mass of 45 g) contains three identical vibration elements, i.e., three small electrodynamic/moving coil cone loudspeakers with spacing of 5 cm among each other (each loudspeaker has diameter of 1 inch, rated power of 3 W, and impedance of 4 Ω). The cone side of each loudspeaker is converted to the contact side with a slightly convex surface (curvature radius 80 mm, constructed by a 3D-printed connector and commercial thin PVC anti-slip mats), and the magnet side is glued to a regular triangular plastic frame. A rough test (at a randomly selected actuation frequency of 350 Hz; see **Supplementary Movies 1** and **2**) indicates that the normal (without additional shear force) and shear (without additional normal force) strengths can be enhanced by 10 and 4 times, respectively, compared with those without vibration.

Supplementary Tables

Supplementary Table 1. Timescale of adhesion switching.

Situation	A_b (μm)	30 to 80	80 to 30	30	40	50
	$\omega/(2\pi)$ (Hz)	450	450	300 to 500	450	450
	$\bar{F}/F_{\mathrm{off,0}}$	68	68	68	37 to 45	6 to 69
Supplementary Figure No.		3(a)-(c)	3(d)-(f)	3(g)-(i)	3(j)-(l)	3(m)-(n)
Switching time (ms)		16	10	33	16	16

Supplementary Table 2. The input power and damping factor at 450 Hz.

A_b (μm)	8.6	23.4	32.3	44.3	56.1	61.9	64.0	76.0	80.5	95.2	96.2	96.5
$F_{\mathrm{off}}/F_{\mathrm{off,0}}$	25.2	61.1	68.2	69.4	67.9	70.4	72.1	65.5	74.8	55.3	74.9	76.8
$P/(\pi r_{\mathrm{eff}}^2)$ (mW mm^{-2}) ($r_{\mathrm{eff}} = 3$ mm)	0.12	1.21	1.25	1.67	1.45	1.83	3.51	1.97	5.57	2.62	4.55	4.91
c (N s m^{-1})	4.09	4.80	6.80	Eq. (21) is invalid								

Supplementary Notes

Supplementary Note 1. Derivation of the governing equation.

Considering an oscillating adhesive contact, both the contact radius a and the penetration depth δ are functions of time t. The contact radius changes due to the dynamic behaviour of a crack (a decrease in a indicates crack growth, and an increase in a indicates crack healing). As is well known, in viscoelastic materials, the effective adhesion work varies with the speed of the moving crack tip [1-6], i.e., $v \approx -\dot{a}$ (an over dot denotes differentiation with respect to time t). To model the dynamic behaviour of the contact interface, the constitutive relation between the apparent adhesion work and the crack propagation speed is required. The concept of apparent (effective) adhesion work was introduced by Schapery [7] and developed by Greenwood, Johnson, Persson, etc. [5, 8-12]. According to Gent's research [13, 14], the relation between w and v is temperature dependent. According to the rate-temperature equivalence principle (WLF, 1955), one can study the relationship at a specific temperature [13] (in this work, the temperature is 23 °C). For small $v(> 0)$, Kendall [15] proposed the relation $w = w_0 + A\,\mathrm{arcsinh}\,Bv$, where w is the apparent (effective) adhesion work, w_0 is the quasi-static (intrinsic) adhesion work, and v is the crack propagation speed. However, the parameters A and B are quite sensitive to material and differ by orders of magnitude for different adhesion systems, which is somewhat inconvenient. At present, for small $v(> 0)$, the widely adopted constitutive relation of the apparent adhesion work is [9, 10, 16-24] $w = w_0[1 + (v/v_0)^{\alpha}]$, where v is the crack propagation speed, v_0 is a positive constant, and α is a constant factor. Theoretically, the fracture theory of viscoelastic materials can be used to study the constitutive relation of apparent adhesion work [8, 11, 12, 25], based on which Greenwood and Johnson predicted that $\alpha = 1/2$ [10]. However, Persson and Brener thought that $\alpha = 1/3$ [17]. The experimentally determined α varies between 0.1 and 0.8. For a large v (such as $v \sim 100$ m/s [26]), an "N" shaped $w \sim v$ curve is generally observed for experimental data [2-4, 6] (Fig. 4). However, the description and mechanism of the constitutive relation at large v values remain a challenge. On the other hand, for $v < 0$, i.e., considering the interface crack healing process [11, 12, 23] Dorogin et al [8] suggested the constitutive relation $w = w_0/(1 + |v/v_0|^{\alpha})$, where $v < 0$. Note that there is still a lack of experimental studies on the apparent adhesion work with $v < 0$. Williamson et al [27] investigated the interface crack healing behaviour based

on the JKR theory [28]. However, such an approach should be improved because the JKR theory itself is not applicable to dynamic adhesion systems.

Similar to the JKR theory, for a dynamic adhesion contact system, it can be assumed that the contact force is repulsive in the contact region and attractive at the edge of the contact region, i.e. the adhesion is valid only at the edge of the contact region. In addition, considering $v \leq v_c$ (v_c is a saturation velocity as marked in Fig. 4), we adopted the adhesive work constitutive relation for the dynamic adhesion contact system with the following form:

$$w(r,v) = \begin{cases} w_0\left(1+\left|\frac{v}{v_0}\right|^{\alpha}\right), & r=a, 0 \leq v \leq v_c, \\ w_0\left(1+\left|\frac{v}{v_0}\right|^{\alpha}\right)^{-1}, & r=a, -\infty < v \leq 0, \\ w_0, & 0 \leq r < a, \\ 0, & r > a, \end{cases} \tag{1}$$

where r is the polar radius (the pole is at the centre of the contact interface), $v = -\dot{a}$; w_0 is the quasi-static (intrinsic) adhesion work of the contact pair; and $v_0 > 0$ is the crack propagation speed, enabling $w = 2w_0$. Eq. (1) and Fig. 4 indicate that the growth and healing of the interface crack have different resistances.

Without loss of generality, we considered a fixed base and a vibrating contactor (Fig. 1b), the kinetic energy of the contactor is $E_k \approx M\dot{\delta}^2/2$, the elastic potential energy of the system is $E_p \approx 3/4 \cdot K(\delta^2 a - 2/3 \cdot \delta a^3/R + a^5/R^2/5)$ [28-30], the bulk dissipation function is $D = c\dot{\delta}^2/2$ (c is the bulk damping coefficient), the generalized force with respect to the generalized coordinate δ is $Q_\delta = F = \bar{F} + f$, and the generalized force with respect to the generalized coordinate a is $Q_a = 2\pi a w(a,v)$, deduced from the elementary work of adhesion $\mathrm{d}W_A = 2\pi a w(a,v)\mathrm{d}a$. According to the Lagrange equations for non-conservative systems [31], i.e.,

$$\begin{cases} \frac{\mathrm{d}}{\mathrm{d}t}\frac{\partial E_k}{\partial \dot{\delta}} - \frac{\partial E_k}{\partial \delta} + \frac{\partial E_p}{\partial \delta} + \frac{\partial D}{\partial \dot{\delta}} = Q_\delta, \\ \frac{\mathrm{d}}{\mathrm{d}t}\frac{\partial E_k}{\partial \dot{a}} - \frac{\partial E_k}{\partial a} + \frac{\partial E_p}{\partial a} + \frac{\partial D}{\partial \dot{a}} = Q_a, \end{cases} \tag{2}$$

one can obtain the governing equation,

$$\begin{cases} \frac{Ka}{2}\left(3\delta - \frac{a^2}{R}\right) + M\ddot{\delta} + c\dot{\delta} = \bar{F} + f, \\ \frac{3K}{8\pi a}\left(\delta - \frac{a^2}{R}\right)^2 = w(a,v), \end{cases} \tag{3}$$

where $\bar{F}$ is the apparent (or average) contact load and $f = A_f \cos \omega t$ (in our experiment, $A_f = MA_b\omega^2$) is the oscillating component of the contact load F.

Supplementary Note 2. Solution of the governing equation when $v \leq v_c$.

When $v \leq v_c$, one can consider the solution in the form of $\delta = \bar{\delta} + \delta_A \cos(\omega t + \varphi)$ ($\delta_A \geq 0$) and $a = \bar{a} + \varepsilon(\omega t)$. $\bar{a}$ is the average value of a, $\bar{\delta}$ is the average value of δ, and ε is a periodic function with respect to ωt. Based on previous work [11, 12] and our experimental results (Fig. 2b), $|\varepsilon|$ is considerably less than $\bar{a}$. Substituting $f = A_f \cos \omega t$ ($A_f \geq 0$), $\delta = \bar{\delta} + \delta_A \cos(\omega t + \varphi)$ and $a = \bar{a} + \varepsilon(\omega t)$ into Eq. (3), we obtain

$$\frac{K\bar{a}}{2}\left(3\delta - \frac{\bar{a}^2}{R}\right) + M\ddot{\delta} + c\dot{\delta} \approx \bar{F} + A_f \cos \omega t, \tag{4a}$$

and

$$\frac{3K}{8\pi\bar{a}}\left(\delta - \frac{\bar{a}^2}{R}\right)^2 \approx \begin{cases} w_0\left(1 + \left|\frac{\dot{\varepsilon}}{v_0}\right|^\alpha\right), & 0 \leq -\dot{\varepsilon} \leq v_c, \\ w_0\left(1 + \left|\frac{\dot{\varepsilon}}{v_0}\right|^\alpha\right)^{-1}, & -\infty < -\dot{\varepsilon} \leq 0. \end{cases} \tag{4b}$$

By averaging Eq. (4a) in the time domain, one can express the apparent contact load $\bar{F}$ as a function of penetration depth $\bar{\delta}$ and average contact radius $\bar{a}$,

$$\bar{F} = \frac{K\bar{a}}{2}\left(3\bar{\delta} - \frac{\bar{a}^2}{R}\right). \tag{5}$$

Substituting Eq. (5) back into Eq. (4a), we have

$$M\ddot{\delta} + c\dot{\delta} = A_f \cos \omega t - \frac{3}{2}K\bar{a}\delta_A \cos(\omega t + \varphi). \tag{6}$$

One can then obtain the steady state solution of Eq. (6):

$$\delta - \bar{\delta} = A_f \frac{(3K\bar{a}/2 - M\omega^2)\cos \omega t + c\omega \sin \omega t}{(3K\bar{a}/2 - M\omega^2)^2 + c^2\omega^2} \triangleq \delta_A \cos(\omega t + \varphi), \tag{7}$$

which yields

$$\delta_A = \frac{A_f}{\sqrt{(3K\bar{a}/2 - M\omega^2)^2 + c^2\omega^2}}. \tag{8}$$

Based on Eq. (4b), $\dot{\varepsilon}$ can be expressed as (for convenience, we let $\varphi = 0$ in the following discussion, which has no effect on the final results)

$$\dot{\varepsilon} = \begin{cases} -v_0|\Psi - 1|^{1/\alpha}, & 0 \leq -\dot{\varepsilon} \leq v_c, \\ v_0|\Psi^{-1} - 1|^{1/\alpha}, & -\infty < -\dot{\varepsilon} \leq 0, \end{cases} \tag{9}$$

where $\Psi = 3K/(8\pi\bar{a}w_0)\cdot(\delta - \bar{a}^2/R)^2$. On the one hand, the continuity of $\dot{\varepsilon}$ at $\dot{\varepsilon} = 0$ requires $|\Psi - 1|^{1/\alpha} = |\Psi^{-1} - 1|^{1/\alpha} = 0$, i.e. $\Psi|_{\dot{\varepsilon}=0} = 1$, or

$$\frac{3K}{8\pi\bar{a}w_0}\left(\bar{\delta} + \delta_A\cos\omega t - \frac{\bar{a}^2}{R}\right)^2 = 1. \tag{10}$$

The solution to Eq. (10) is

$$t_0 = \frac{2k\pi}{\omega} \pm \frac{\tau}{\omega}\ (k \text{ is an integer}), \tag{11}$$

where $\tau = \arccos\left[1/\delta_A\cdot\left(\bar{a}^2/R - \bar{\delta} - \sqrt{8/3\cdot\pi\bar{a}w_0/K}\right)\right]$, and $\left|\bar{a}^2/R - \bar{\delta} - \sqrt{8/3\cdot\pi\bar{a}w_0/K}\right| \le \delta_A$ should be satisfied to avoid imaginary solutions. On the other hand, the continuity of a requires that

$$v_0\int_{\tau/\omega}^{(2\pi-\tau)/\omega}|\Psi - 1|^{1/\alpha}\mathrm{d}t = v_0\int_{-\tau/\omega}^{\tau/\omega}\left|\frac{1}{\Psi} - 1\right|^{1/\alpha}\mathrm{d}t. \tag{12}$$

The left-hand side of Eq. (12) is the total decrement in the contact radius due to crack growth, and the right-hand side is the total increment in the contact radius due to crack healing. $\varepsilon \ll \bar{a}$, so $\int_{\tau/\omega}^{(2\pi-\tau)/\omega}|\Psi - 1|^{1/\alpha}\mathrm{d}t \ll \bar{a}$. With Eq. (12), $\bar{a}$ can be solved as a function of $\bar{\delta}$ and δ_A. Eq. (12) can be solved numerically. To find an approximate solution, one can notice from Eq. (9) that $\bar{a}$ is not related to ω, and let $\omega \to 0$; finally, Eq. (12) reveals that

$$\bar{\delta} \to \frac{\bar{a}^2}{R} - \delta_A - \sqrt{\frac{8\pi\bar{a}w_0}{3K}}. \tag{13}$$

Notably, Eq. (13) includes the limit of $\tau \to 0$, which indicates an infinite crack healing speed and $w(a, v) \to 0 (v < 0)$. Such an approximation holds only when the actuation frequency is high enough. Note that $\omega \to 0$ indicates infinite displacement, which is only theoretically feasible. Thus, ω should be large enough, i.e., $\omega \gg 0$, in actual estimation or theoretical prediction. Substituting Eqs. (8) and (13) into Eq. (5), the apparent contact load (i.e., Eq. (3)) can be obtained as

$$\bar{F}(A_f, \omega, \bar{a}) = \frac{K\bar{a}^3}{R} - \sqrt{6\pi w_0 K\bar{a}^3} - \frac{3}{2}\frac{A_f K\bar{a}}{\sqrt{(3K\bar{a}/2 - M\omega^2)^2 + c^2\omega^2}}, \tag{14}$$

where $A_f \ge 0$, $\omega \gg 0$, and $v \le v_c$. Eq. (14) can also be expressed as the function of the input power,

$$\bar{F} = \frac{K\bar{a}^3}{R} - \sqrt{6\pi w_0 K\bar{a}^3} - \frac{3K\bar{a}}{\omega}\sqrt{\frac{P}{2c}}, \tag{15}$$

where $P = \omega/(2\pi) \cdot \oint f \mathrm{d}\delta = c\omega^2/2 \cdot A_f^2/[(3K\bar{a}/2 - M\omega^2)^2 + c^2\omega^2]$ is the input power (Eq. (7) is considered). Eq. (15) reveals a simple relation among $\bar{F}$, and P and ω.

In summary, Eqs. (5), (8), and (13) and the fact that ε can be obtained by integrating Eq. (9) with respect to time t provides the full solution to Eq. (3), i.e., Eq. (1).

Supplementary Note 3. Discussion of the pull-off force.

By determining the minimum value of $\bar{F}$ for a given combination of A_f and ω, the pull-off force F_{off} can be obtained based on Eq. (14),

$$F_{\text{off}} = \min\{\bar{F}\} \approx \begin{cases} F_{\text{off},0}, & A_f \to 0, \\ -A_f\sqrt{1 + M^2\omega^2/c^2}, & A_f \gg 0, \end{cases} v \le v_c, \tag{16}$$

where $F_{\text{off},0} = -3\pi R w_0/2$ is the quasi-static pull-off force predicted by the JKR theory. The minimum value $-A_f\sqrt{1 + M^2\omega^2/c^2}$ can be determined when $\bar{a} = 2(c^2 + M^2\omega^2)/(3KM)$. In addition, by letting $\max\{v\} \le v_c$ and referring to Eqs. (8) and (9), one can find that $\max\{v\} \le v_c$ is equivalent to

$$A_f \le A_{fc} = \sqrt{\left(\frac{3K\bar{a}}{2} - M\omega^2\right)^2 + c^2\omega^2}\sqrt{\frac{2\pi\bar{a}w_0}{3K}}\left(\sqrt{1 + \left|\frac{v_c}{v_0}\right|^\alpha} - 1\right). \tag{17}$$

$A_f \le A_{fc}$ describes the restrictions on the actuation forces to ensure $v \le v_c$. In addition, for a given combination of A_f and ω, $A_f = A_{fc}$ gives the solution of $\bar{a} = \bar{a}_c$.

For a given combination of A_f and ω that satisfies $\max\{v\} \ge v_c$, the system could lose stability because of the rapid drop in effective adhesive work (Fig. 4), and the apparent adhesive force may decrease or even disappear; in such cases, the excessive predictions are provided by Eq. (16). Thus, when $\max\{v\} \gg v_c$, $\bar{F} \to 0$. For a $\max\{v\}$ that is not considerably greater than v_c, by substituting Eq. (17) into Eq. (14), one can find $F_{\text{off}} \approx \min\{\bar{F}(A_f, \omega, \bar{a})\} \approx |v_c/v_0|^\alpha F_{\text{off},0}/4$ when $\bar{a} \approx \sqrt[3]{3/8 \cdot \pi w_0 R^2/K \cdot |v_c/v_0|^\alpha}$. Otherwise, F_{off} should be between $|v_c/v_0|^\alpha F_{\text{off},0}/4$ and the value of $\bar{F}$ when $\bar{a} = \bar{a}_c$ ($\bar{a}_c$ is not unique), i.e. $|v_c/v_0|^\alpha F_{\text{off},0}/4 \le F_{\text{off}} \le \min\{\bar{F}(A_f, \omega, \bar{a}_c)\}$. Finally, we have

$$F_{\text{off}} \approx \begin{cases} 0, & \max\{v\} \gg v_c, \\ \frac{1}{4}\left|\frac{v_c}{v_0}\right|^{\alpha} F_{\text{off},0}, & \max\{v\} \sim v_c, \\ \left[\frac{1}{4}\left|\frac{v_c}{v_0}\right|^{\alpha} F_{\text{off},0}, \min\{\bar{F}(A_f, \omega, \bar{a}_c)\}\right], & \text{Otherwise,} \end{cases} \quad \max\{v\} \geq v_c, \tag{18}$$

where we consider $v_c \gg v_0$.

Eqs. (16) and (18) indicate that the pull-off force satisfies

$$0 \leq \frac{F_{\text{off}}}{F_{\text{off},0}} \leq \frac{1}{4}\left|\frac{v_c}{v_0}\right|^{\alpha}. \tag{19}$$

Supplementary Note 4. The effect of the platform thickness on contact behaviour.

Considering a typical contact load as 60% of the minimum pull-off force in our experiment, i.e., $\bar{F} \approx -0.4$ N, one can consider a Hertz contact with $F = 0.4$ N, which possesses a similar local effect since the local effect is almost independent of the sign of F for a linear elastic system. For a typical simulation on Hertz contact under the conditions of $R = 51.64$ mm, $K = 6.211$ MPa, and PDMS base thickness of 3 mm, the contact stiffness (proportional to K) is 2.23 times that for the ideal Hertz contact with an infinite base thickness. The simulated results are shown in **Supplementary Figure 1**.

Supplementary Note 5. Timescale of adhesion switching.

Generally, the plot of the displacement of the contactor (δ, vertical axis) versus the displacement of the platform ($A_b \cos\omega t$, horizontal axis) shows a series of loops (**Supplementary Figure 2**). The loops under one vibration state plot close together. Thus, two vibration states should have two clusters of loops. One can observe two distinguishable clusters of loops in **Supplementary Figure 2**(g)-(n). Although the two clusters of loops may overlap (e.g., in **Supplementary Figure 2**(a)-(f)), clearly, ungrouped loops representing the adhesion switching processes can be identified in all the considered cases. Thus, the number of ungrouped loops (N) can be used to estimate the switching time. Specifically, in **Supplementary Figure 2**(a)-(c), the average value of N is 21/3, and the switching time is thus $N \cdot 2\pi/\omega \approx 16$ ms. Similarly, the switching times for **Supplementary Figure 2**(d)-(f), **Supplementary Figure 2**(g)-(i), **Supplementary Figure 2**(j)-(l), and **Supplementary Figure 2**(m)-(n) are $14/3 \cdot 2\pi/\omega \approx 10$ ms, $50/3 \cdot 2\pi/\omega \approx 33$ ms ($\omega/(2\pi)$ taken as 500 Hz), 22/3$\cdot$ $2\pi/\omega \approx 16$

ms, and $14/2 \cdot 2\pi/\omega \approx 16$ ms, respectively (see **Supplementary Table 1** for details). Alternatively, when the apparent contact load is abruptly changed, such as from $\bar{F}/F_{\mathrm{off},0} = 6$ to 95 (approximately 120% of the maximum theoretical value) under an actuation frequency of 450 Hz and amplitude of 50 μm, and the adhesion is maintained for approximately 1 s, which may be long enough for some extreme working conditions.

Supplementary Note 6. Energy consumption analysis.

As defined in Eq. (15), the input power can be calculated as

$$P = \frac{\omega}{2\pi} \oint f \mathrm{d}\delta. \tag{20}$$

Eq. (20) can be used to calculate the actual power based on the experimental data by integrating within the internal area of a closed loop of the $A_b \cos \omega t \sim \delta$ curves (similar to the those in **Supplementary Figure 2**). Theoretically, it is readily obtained that (when $v \leq v_c$)

$$P = \frac{c\omega^2}{2} \frac{A_f^2}{(3K\bar{a}/2 - M\omega^2)^2 + c^2\omega^2}. \tag{21}$$

Eq. (21) can be used to estimate the damping factor c. A group of data of the input power and the damping factor are listed in **Supplementary Table 2**, where $P/(\pi r_{\mathrm{eff}}^2)$ represents the required input power per unit contact area and is experimentally determined based on Eq. (20) and the corresponding recorded displacement data.

Supplementary References

1 Barthel, E. Adhesive elastic contacts: JKR and more. *J. Phys. D-Appl. Phys.* **41**, 163001 (2008).
2 Creton, C. & Ciccotti, M. Fracture and adhesion of soft materials: a review. *Rep. Prog. Phys.* **79**, 046601 (2016).
3 Dalbe, M.J., Santucci, S., Cortet, P.P. & Vanel, L. Strong dynamical effects during stick-slip adhesive peeling. *Soft Matter* **10**, 132-138 (2014).
4 Dalbe, M.J. et al. Inertial and stick-slip regimes of unstable adhesive tape peeling. *Soft Matter* **12**, 4537-4548 (2016).
5 Tiwari, A. et al. The effect of surface roughness and viscoelasticity on rubber adhesion. *Soft Matter* **13**, 3602-3621 (2017).
6 Villey, R. et al. Rate-dependent elastic hysteresis during the peeling of pressure sensitive adhesives. *Soft Matter* **11**, 3480-3491 (2015).
7 Schapery, R.A. On the mechanics of crack closing and bonding in linear viscoelastic media. *Int. J. Fracture* **39**, 163-189 (1989).
8 Dorogin, L., Tiwari, A., Rotella, C., Mangiagalli, P. & Persson, B.N.J. Role of Preload in Adhesion of Rough Surfaces. *Phys. Rev. Lett.* **118**, 238001 (2017).
9 Greenwood, J.A., Johnson, K.L. The mechanics of adhesion of viscoelastic solids. *Philos. Mag. A* **43**, 697-711 (1981).
10 Persson, B.N.J. & Brener, E.A. Crack propagation in viscoelastic solids. *Phys. Rev. E* **71**, 036123 (2005).
11 Greenwood, J.A. & Johnson, K.L. Oscillatory loading of a viscoelastic adhesive contact. *J. Colloid Interface Sci.* **296**, 284-291 (2006).
12 Wahl, K.J., Asif, S.A.S., Greenwood, J.A. & Johnson, K.L. Oscillating adhesive contacts between micron-scale tips and compliant polymers. *J. Colloid Interface Sci.* **296**, 178-188 (2006).
13 Gent, A.N., Ptrich, R.P. Adhesion of viscoelastic materials to rigid substrates. *Proc. R. Soc. A-Math. Phys. Eng. Sci.* **310**, 433-448 (1969).
14 Gent, A.N., Schultz, J. Effect of wetting liquids on the strength of adhesion of viscoelastic materials. *The Journal of Adhesion* **3**, 281-294 (1972).
15 Kendall, K. Peel adhesion of solid films-The surface and bulk effects. *The Journal of Adhesion* **5**, 179-202 (1973).
16 Barthel, E. & Perriot, A. Adhesive contact to a coated elastic substrate. *J. Phys. D-Appl. Phys.* **40**, 1059-1067 (2007).
18 11
17 Muller, V.M. On the theory of pull-off of a viscoelastic sphere from a flat surface. *J. Adhes. Sci. Technol.* **13**, 999-1016 (1999).
18 Barthel, E. & Roux, S. Velocity-dependent adherence: An analytical approach for the JKR and DMT models. *Langmuir* **16**, 8134-8138 (2000).
19 Maugis, D. & Barquins, M. Fracture mechanics of adhesion of viscoelastic bodies. *J. Phys. D-Appl. Phys.* **11**, 1989-2023 (1978).
20 Sun, T.L. et al. Bulk energy dissipation mechanism for the fracture of tough and self-healing hydrogels. *Macromolecules* **50**, 2923-2931 (2017).
21 Greenwood, J.A. The theory of viscoelastic crack propagation and healing. *J. Phys. D-Appl. Phys.* **37**, 2557-2569 (2004).
22 Greenwood, J.A. Viscoelastic crack propagation and closing with Lennard-Jones surface forces. *J. Phys. D-Appl. Phys.* **40**, 1769-1777 (2007).
23 Greenwood, J.A., Johnson K.L., Choi S.H., and Chaudhury M.K. *J. Phys. D-Appl. Phys.* **42**, 035301 (2009).
24 Shull, K.R. Contact mechanics and the adhesion of soft solids. *Mater. Sci. Eng. R-Rep.* **36**, 1-45 (2002).
25 Barthel, E. & Fretigny, C. Adhesive contact of elastomers: effective adhesion energy and creep function. *J. Phys. D-Appl. Phys.* **42**, 195302 (2009).
26 Cortet, P.P., Ciccotti, M., Vanel, L. Imaging the stick-slip peeling of an adhesive tape under a constant load. *J. Stat. Mech.-Theory Exp.* **3**, P03005 (2007).
27 Williamson, D. M., Hamilton, N. R., Jardine, A. P. Rate dependent interfacial properties using the JKR experimental technique. *Proceedings of the 2016 Annual Conference on Experimental and Applied Mechanics*, **2** 49-54 (2017).
28 Johnson, K.L., Kendall, K. & Roberts, A.D. Surface energy and the contact of elastic solids. *Proc. R. Soc. A-Math. Phys. Eng. Sci.* **324**, 301-303 (1971).
29 Popov, V.L. Contact Mechanics and Friction: Physical Principles and Applications (2010). Springer-Verlag, Berlin, Heidelberg.
30 Johnson, K.L. Contact Mechanics (1985). Cambridge University Press.
31 Wang, Y.G. Analytical Mechanics (2019) (In Chinese). Tsinghua University Press.
