# Supplementary material for: Rapid and continuous regulating adhesion strength by mechanical micro-vibration
Source: Nat Commun. 2020 Mar 27;11:1583. doi: 10.1038/s41467-020-15447-x (PMC7101336; doi:10.1038/s41467-020-15447-x)
Supplement: Supplementary file 3 — Description of Additional Supplementary Files [file 41467_2020_15447_MOESM3_ESM.pdf]

Description of Additional Supplementary Files

Supplementary Movie 1.

Regulation of the interface adhesion between a flat glass and a vibrating soft contactor without shear force. The details refer to Supplementary Figure 3.

Supplementary Movie 2.

Regulation of the interface adhesion between a flat glass and a vibrating soft contactor with shear force. The details refer to Supplementary Figure 3.
